# Factors affecting the outcome of lower extremity osteomyelitis treated with microvascular free flaps: an analysis of 65 patients

**DOI:** 10.1186/s13018-021-02686-x

**Published:** 2021-08-27

**Authors:** Duy Quang Thai, Yeon Kyo Jung, Hyung Min Hahn, Il Jae Lee

**Affiliations:** 1 Department of Plastic and Reconstructive Surgery, Ajou University School of Medicine, 164 World cup-ro, Yeongtong-gu, Suwon-si, Gyeonggi-do 16499 Republic of Korea; 2 Department of Plastic and Reconstructive Surgery, Hanoi Medical University, 1 Ton That Tung, Kim Lien, Dong Da, Hanoi, Vietnam

**Keywords:** Osteomyelitis, Free flaps, Diabetic foot ulcer, Peripheral arterial occlusive disease

## Abstract

**Background:**

Free flaps have been a useful modality in the management of lower extremity osteomyelitis particularly in limb salvage. This study aimed to determine the factors affecting the outcome of free flap reconstruction in the treatment of osteomyelitis.

**Methods:**

This retrospective study assessed 65 osteomyelitis patients treated with free flap transfer from 2015 to 2020. The treatment outcomes were evaluated in terms of the flap survival rate, recurrence rate of osteomyelitis, and amputation rate. The correlation between outcomes and comorbidities, causes of osteomyelitis, and treatment modalities was analyzed. The following factors were considered: smoking, peripheral artery occlusive disease, renal disease, diabetic foot ulcer, flap types, using antibiotic beads, and negative pressure wound therapy.

**Result:**

Among the 65 patients, 21 had a severe peripheral arterial occlusive disease. Osteomyelitis developed from diabetic foot ulcers in 28 patients. Total flap failure was noted in six patients, and osteomyelitis recurrence was noted in eight patients, for which two patients underwent amputation surgery during the follow-up period. Only end-stage renal disease had a significant correlation with the recurrence rate (odds ratio = 16.5, *p* = 0.011). There was no significant relationship between outcomes and the other factors.

**Conclusion:**

This study showed that free flaps could be safely used for the treatment of osteomyelitis in patients with comorbidities and those who had osteomyelitis developing from diabetic foot ulcers. However, care should be taken in patients diagnosed with end-stage renal disease.

## Background

Osteomyelitis (OM) has been one of the most difficult complications. Patients with OM have to face the possibility of amputation that can adversely affect their quality of life [1]. The treatment of OM aims to eradicate infection, obliterate dead space, and cover soft-tissue defects. Over the last three decades, free tissue transfer has been an important option that can overcome the limitations of typical methods such as skin grafts, local flaps, or muscle flaps. Free flaps can provide a wide range of tissues including skin, muscle, bone, or a combination of these to allow for more aggressive resection of affected tissues. Consequently, this decreases the recurrence rate of osteomyelitis and increases the rate of limb salvage. Numerous studies have reported favorable results of using free flaps in the treatment of osteomyelitis [2–4]. However, to the best of our knowledge, factors affecting the operative results have not been well established. Therefore, we aimed to determine whether co-morbidities and other factors could influence the outcome of using free flaps in the treatment of lower extremity osteomyelitis.

## Patients and methods

This retrospective study was approved by the Institutional Review Board of our medical center (AJIRB-MED-MDB-16-402) and we assessed patient records from 2015 to 2020. Sixty-five patients diagnosed with osteomyelitis and treated with free flaps were included. The average follow-up period was 18 months (range, 6 to 48 months). Patients with incomplete reports or those who followed up for less than 6 months were excluded. Patients under 16 years of age were also excluded from this study.

The following data was collected via patient charts: demography, radiographs of the lower extremity, details of the wounds and treatment, complications, and follow-up information. The outcomes of treatment were evaluated based on flap success rate, recurrence rate of OM, and amputation rate during the follow-up period. The correlation between outcomes and comorbidities, causes of osteomyelitis, and treatment modalities were analyzed.

### Statistical analysis

Categorical variables were summarized as frequencies and percentages while continuous variables were expressed as mean and standard deviations. Chi-square and Fisher’s exact tests were used to analyze the difference in flap survival rate and the recurrence rate of osteomyelitis in patients with and without the following factors: smoking, peripheral artery occlusive disease (PAOD), end-stage renal disease (ESRD), diabetic foot ulcer, flap types, using antibiotic beads, and negative pressure wound therapy (NPWT). A *p* value < 0.05 was considered statistically significant. All statistical analyses were performed using SPSS 20.0. (IBM Corp., Armonk, NY, USA).

### Treatment protocol

The diagnosis of osteomyelitis was based on clinical symptoms; laboratory tests including WBC, ESR, CRP, tissue culture, and bone culture; and radiological images including plain X-rays, MRI, and bone scans. Simultaneously, patients were thoroughly examined for cardiac, endocrine, and renal conditions. These conditions were managed by specialist physicians whenever necessary. Lower extremity blood vessel status was also carefully evaluated. If CT-angiography was unclear, it was supplemented with traditional angiography. All of the patients with severe stenosis (greater than 75% of the diameter) or total occlusion underwent percutaneous transluminal angioplasty. Serial debridement was performed to radically resect the infected tissues including soft tissue and bone. Deep soft tissue and bone cultures were taken to confirm the diagnosis and direct the antibiotic treatment. Patients received broad-spectrum antibiotic treatment before culture results, and specific antibiotics following the confirmation of culture results. Antibiotic beads (Vancomycin) were used in 15 cases and NPWT was applied in 44 cases.

Reconstruction was performed once all the infected tissues were resected and the wound had a good blood supply. The operation began with appropriate debridement followed by dissection of the recipient vessels. Major arteries with higher blood pressure were preferred over small branches as the recipient vessels. These branches were preserved to prevent further damage. Arteriorrhaphy was attempted in all cases with an end-to-side approach; an end-to-end anastomosis was performed if the end-to-side approach was unsuccessful. The type of flap was decided according to the size and depth of the defect. Although the fasciocutaneous flap was most commonly used, myocutaneous flaps were selected to obliterate dead space in cases with deep defects.

### Case reports

#### Case 1

**Fig. 1:**
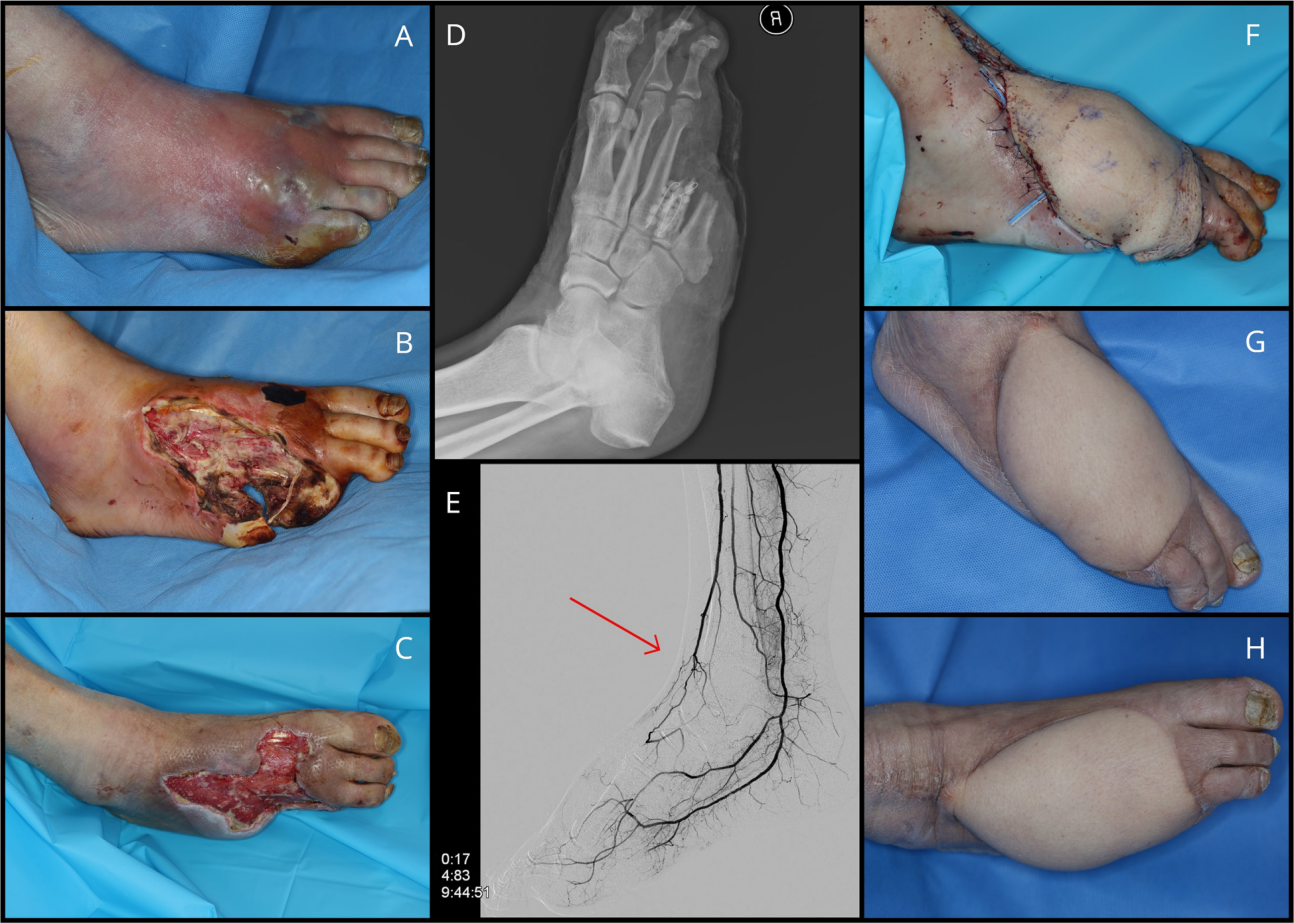
Case 1. **A** Diabetic foot infection. **B**, **C** Ischemic defect following serial debridement. **D** Antibiotic beads insertion. **E** Angiography showing the total occlusion of the dorsalis pedis artery (red arrow). **F** Coverage the defect with fasciocutaneous ALT free flap. **G**, **H** 8 months post-operation

A 56-year-old male diabetic patient was referred to our department with diabetic foot osteomyelitis. He had been hypertensive for 20 years and diabetic for 24 years. CT-angiography revealed severe stenosis of the popliteal artery and total occlusion of the dorsalis pedis artery of his right leg. Immediate balloon angioplasty was performed. Serial debridement and minor amputation were done—his fourth and fifth toes were resected. A fasciocutaneous anterolateral thigh free flap (ALT) was applied to completely cover the defect with antibiotic beads inserted underneath. Due to atherosclerosis, the anastomosis was performed in an end-to-side fashion to the anterior tibial artery proximal to the occlusion. The flap healed uneventfully and the patient was ambulatory during 8 months of follow-up (Fig. 1).

#### Case 2

**Fig. 2:**
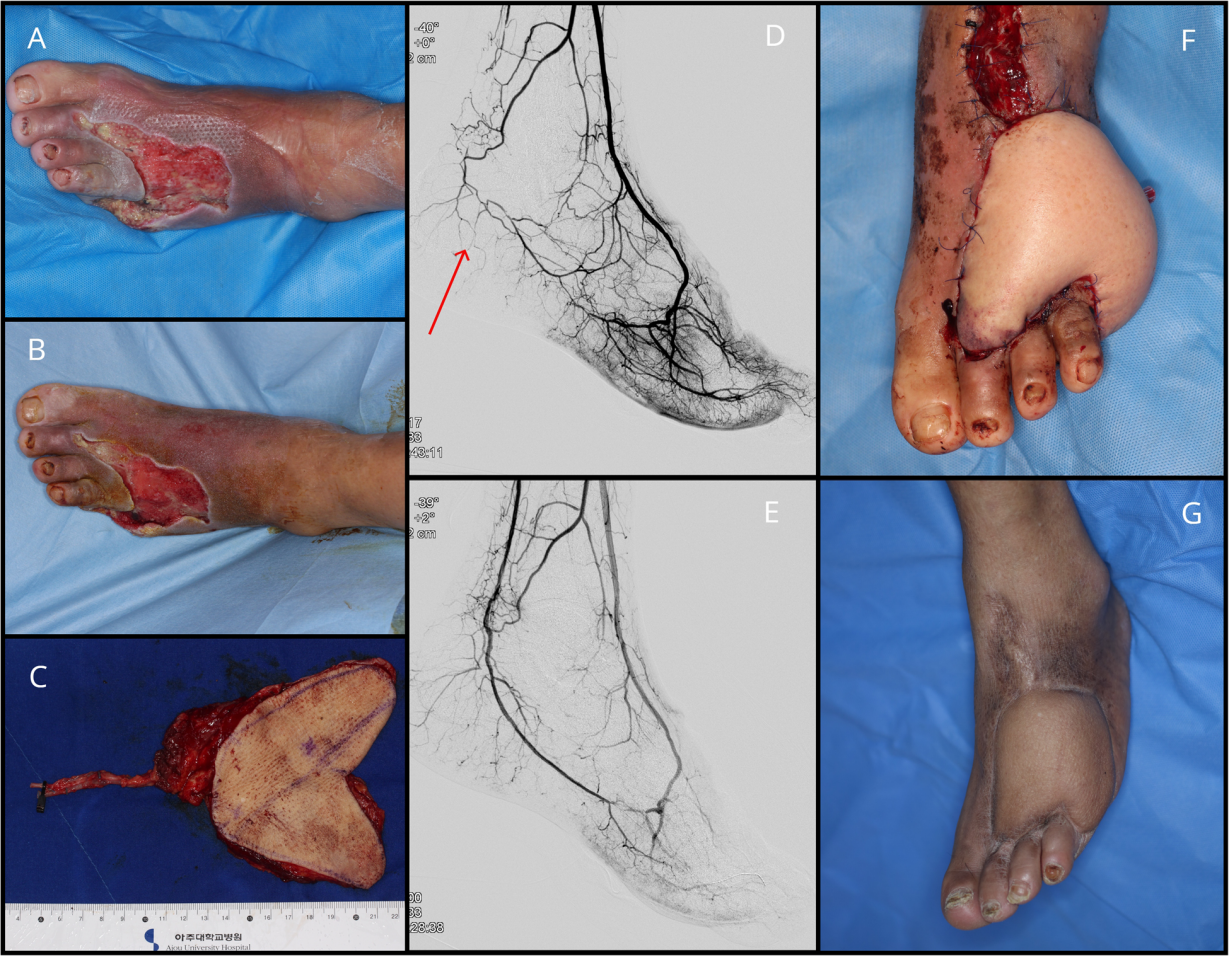
Case 2. **A**, **B** Diabetic foot ulcer after debridement and NPWT. **C** Myocutaneous ALT free flap. **D**, **E** Before and after images of successful angioplasty of posterior tibial artery. **F** Two days post-operation. **G** 41 months post-operation

A 55-year-old man, diabetic for 20-years, was admitted for the development of wet gangrene in his fifth toe. The toe was resected, and serial debridement was performed. Preoperative angioplasty indicated the absence of blood flow from the posterior tibial artery to the pedal arch due to total occlusion. He underwent balloon angioplasty of the left posterior tibial artery and plantar artery. After angioplasty, a complete pedal arch was seen with the supply from both the dorsalis pedis artery and the plantar artery. A myocutaneous ALT flap was designed and harvested based on the size of the defect. The flap was then anastomosed to the anterior tibial artery in an end-to-side manner. No additional surgery was required. Forty-one months of follow-up revealed a well-healed, well-perfused free flap without any sign of osteomyelitis recurrence (Fig. 2).

## Results

Patient demographics are listed in Table 1. A total of 65 patients with lower leg osteomyelitis were treated with microvascular free flaps. The mean age was 51.2 ± 15.8 (range, 18 to 82 years), with 49 male patients and 16 female patients. Twenty-four patients were active smokers. Thirty-one patients had diabetes, 21 had severe obstruction of lower extremity arteries, which needed angioplasty, and five patients had ESRD. The defects were most commonly located in the foot (43.1%), followed by the tibia, heel, and ankle (14 patients, 13 patients, and 6 patients, respectively). Twenty-nine patients developed osteomyelitis following trauma, 28 patients had diabetic foot ulcer osteomyelitis. The other causes were pressure sore, burn, skin infection.

**Table 1:** Patient demographics

Characteristics		No of patients (%)
Age		51.17 ± 15.83
BMI		23.58 ± 3.68
Gender	Male	49 (75.4%)
	Female	16 (24.6%)
Diabetes	No	34 (52.3%)
	Yes	31 (47.7%)
Wound location	Knee	2 (3.1%)
	Tibia	14 (21.5%)
	Ankle	6 (9.2%)
	Heel	13 (20%)
	Foot	28 (43.1%)
	Multiple	2 (3.1%)
Cause of wound	Trauma	29 (44.6%)
	Diabetic foot ulcer	28 (43.1%)
	Others	8 (12.3 %)

The ALT flap was most commonly used (92.3%) followed by three medial sural artery perforator flaps, one superficial circumflex iliac perforator flap, and one thoracodorsal artery perforator flap. Out of 65 cases, total flap loss was seen in six patients (9.23%). Three of the six flap failures were successfully replaced with new fasciocutaneous free flaps. The other three patients underwent additional debridement with split-thickness skin grafts or NPWT and secondary healing.

Recurrence of OM was seen in eight patients (12.3%). Amongst these, two patients underwent below the knee amputation, one patient underwent another ALT free flap surgery and five patients were treated with minor debridement and oral antibiotics. Both patients who underwent major amputation suffered from serious medical conditions with diabetic foot ulcers, ESRD, and severe occlusion of the lower limb arteries. Although they successfully underwent angioplasty, one patient presented a total flap necrosis then he was treated with NPWT and secondary healing. Their osteomyelitis recurred within three months of primary treatment.

A significant difference in recurrence rate was found in patients with ESRD and those without ESRD (*p* = 0.011, OR = 16.5). PAOD and diabetic foot ulcers had no correlation with flap survival as well as recurrence rate. Similarly, other factors had no significant correlation with the outcomes. Although the association was not significant, there was a tendency of reducing recurrence in patients treated using antibiotic beads and NPWT (OR = 0.439 and 0.769, respectively) (Table 2).

**Table 2:** Analysis of factors affecting the results of treatment

Factors		Flap loss				Recurrence			
		No	Yes	OR	*p*	No	Yes	OR	*p*
Smoking	No	37	4	0.841	0.611	36	5	1.029	0.628
	Yes	22	2			21	3		
PAOD	No	40	4	1.053	0.638	40	4	2.353	0.42
	Yes	19	2			17	4		
Diabetic foot ulcer	No	34	3	1.360	0.522	33	4	1.375	0.717
	Yes	25	3			24	4		
ESRD	No	55	5	2.750	0.394	55	5	16.5	0.011^a^
	Yes	4	1			2	3		
Flap type	Fasciocutaneous					48	6	1.778	0.614
	Myocutaneous					9	2		
Antibiotic beads	No					43	7	0.439	0.669
	Yes					14	1		
NPWT	No					18	3	0.769	0.706
	Yes					39	5		

^a^Statistical significant

## Discussion

Although the diagnosis and treatment of osteomyelitis has improved, it remains a significant challenge to physicians. Management of osteomyelitis involves using antibiotics, radical debridement, and reconstruction with well-vascularized tissue. Several studies reported the efficacy of using free tissue transfer in the treatment of chronic osteomyelitis [5–10]. Free flaps regardless of the composition provide reliable tissues not only to cover large defects but also to obliterate dead space, without further compromising local tissue [2, 11]. In this study, the authors aimed to evaluate the influence of co-morbidities and other factors such as diabetic foot ulcers, flap types, using antibiotic beads and NPWT on the outcome of osteomyelitis treated with free flaps. The only factor that had a significant correlation with recurrence rate was ESRD. Patients with ESRD had 16.5 times higher odds of OM recurrence (*p* = 0.011). Renal failure has been known to impair wound healing in several ways [12, 13]. Additionally, it has been considered as a risk factor for free flap failure and limb amputation [14–16]. Therefore, caution must be taken when treating OM in patients with ESRD and free flaps should be carefully monitored post-operation.

PAOD is closely related to diabetes and smoking. Atherosclerotic changes decrease blood supply to the distal parts of the extremities. Consequently, it compromises the wound healing process and increases the rate of flap failure. Lee et al. reported a high rate of flap loss when they performed a combination of revascularization and free flap for severe PAOD patients [16]. Similarly, Oh et al. reported 10.212 times higher odds of flap failure in PAOD patients [17]. Nevertheless, our previous study showed the safe utilization of free flaps in PAOD patients following a successful angioplasty, particularly in cases where angioplasty achieved the complete revascularization of the pedal arch [18]. Similarly, in this study, all 21 patients with severely stenotic arteries underwent angioplasty before flap surgery, and complete flap necrosis was only seen in two cases. We believe that maximizing the blood flow by arterial intervention played a critical role in the reconstruction of ischemic wounds of the lower extremities.

Osteomyelitis following diabetic foot ulcers is a critical condition associated with complications and a high rate of lower limb amputation [19]. Choosing conservative or surgical treatment for diabetic foot osteomyelitis has been debated for years; however, the management of diabetic foot osteomyelitis still varies from center to center [1, 20–22]. In our institute, we believe that the combination of surgical and medical treatment would provide the better result. Although radical debridement often results in a large defect, this defect is frequently able to obliterate by the free flap transfer. With this in mind, the surgeon can completely resect the infected and unviable tissue including skin, soft tissue, and bone without any hesitation. The result of our study showed no statistically significant difference of the outcomes between the group with diabetic foot ulcer and those with non-diabetic foot ulcer. Other studies also revealed similar results with a relatively high rate of limb salvage, which was very encouraging for the treatment of osteomyelitis secondary to a diabetic foot [17, 23].

Besides the typical treatment modalities, adjuvant therapies including antibiotic beads and NPWT have been proven effective in the management of OM [24–26]. Data analysis revealed that there was a tendency of reduced recurrence in patients who used antibiotic beads and NPWT. However, it was not statistically significant.

This study has several limitations since it was a single-center, retrospective, non-randomized study with a small sample size. Additionally, the small number of patients who underwent major amputation (two patients) was the reason we could not perform statistical analysis in this group. However, it revealed a high rate of limb salvage (96.9%) comparable to other studies. The relatively short duration of follow-up was the other limitation, as osteomyelitis can recur several years after treatment. Hence, further investigation should be continued in these patients. Despite these limitations, we believe that this study showed the efficacy of free flaps in the treatment of osteomyelitis and limb salvage even in patients with co-morbidities.

## Conclusion

This study found patients with ESRD had a higher rate of recurrence of osteomyelitis after treatment. The other factors had no significant correlation with the outcomes. Free flap coverage for OM patients is a reliable approach to achieve successful remission and limb salvage. The keystones of treatment are to eradicate infections, cover wounds with well-vascularized tissue and simultaneously control comorbid conditions. Besides, maximizing the distal blood supply of the lower extremity essentially contributes to enhancing the success rate of the treatment, especially in patients with severe PAOD.

## Data Availability

Please contact the corresponding author for data request.

## Abbreviations

OM: Osteomyelitis
PAOD: Peripheral arterial occlusive disease
ESRD: End-stage renal disease
NPWT: Negative pressure wound therapy
ALT: Anterolateral thigh
